# Development and Structure of Internal Glands and External Glandular Trichomes in *Pogostemon cablin*

**DOI:** 10.1371/journal.pone.0077862

**Published:** 2013-10-30

**Authors:** Jiansheng Guo, Yongming Yuan, Zhixue Liu, Jian Zhu

**Affiliations:** 1 Department of Molecular and Cell Biology, School of Life Science and Technology, Tongji University, Shanghai, China; 2 Firmenich Company, Shanghai, China; Wuhan University, China

## Abstract

*Pogostemon cablin* possesses two morphologically and ontogenetically different types of glandular trichomes, one type of bristle hair on the surfaces of leaves and stems and one type of internal gland inside the leaves and stems. The internal gland originates from elementary meristem and is associated with the biosynthesis of oils present inside the leaves and stems. However, there is little information on mechanism for the oil biosynthesis and secretion inside the leaves and stems. In this study, we identified three kinds of glandular trichome types and two kinds of internal gland in the *Pogostemon cablin*. The oil secretions from internal glands of stems and leaves contained lipids, flavones and terpenes. Our results indicated that endoplasmic reticulum and plastids and vacuoles are likely involved in the biosynthesis of oils in the internal glands and the synthesized oils are transported from endoplasmic reticulum to the cell wall via connecting endoplasmic reticulum membranes to the plasma membrane. And the comparative analysis of the development, distribution, histochemistry and ultrastructures of the internal and external glands in *Pogostemon cablin* leads us to propose that the internal gland may be a novel secretory structure which is different from external glands.

## Introduction

The Lamiaceae comprise many commercially important species because of their high content of essential oils, which are widely used in pharmaceutical preparations, perfumery and cosmetics. The development and histochemistry of glandular trichomes occurring in plants of the Lamiaceae was well documented [1]–[6] and they were recognized as the defense-related structures on the aerial epidermis of leaves, stems and floral organs [7], [8]. As to the types of trichomes (capitate, non-glandular, peltate and different versions and combinations of these), there was some variability in Lamiaceae genera that can occur in a given species [9], [10]. And each type of trichomes had a different spatial arrangement and function, secreting different combinations, or proportions, of hydrophilic and lipophilic material.

Most published studies on the Lamiaceae had concentrated on the ultrastructure of peltate trichomes or capitate trichomes in relation to the secretory process [11]–[16]. Basing upon the remarkable ultrastructural transformations occurring at the onset of secretion in the glandular trichomes, previous investigators proposed a range of speculations concerning the possible sites of oil synthesis and the possible mechanisms of oil secretion. There is much distinction about the ultrastructure and secretory process in different glandular trichomes of different species.

*Pogostemon cablin* is one of the tropical, aromatic crops in Lamiaceae, and cultivated mainly in Southeast Asia, India and Brazil. Previous studies on *Pogostemon cablin* have shown that there are external or internal glands in leaves and stems [17], [18]. These structures are responsible for the production of sesquiterpenes containing a large quantity of patchouli alcohol, of which more than 30 percent of the essential oils [19], [20]. However, little is known about secretory process and the composition of the secreted material in internal glands of leaves and stems. And the distinction about the ultrastructure, secretory process and development among the glands of *Pogostemon cablin* deserves to be studied for elucidate the relationship between the external and internal glands.

## Results

### Development, Histochemistry and Secretion of Glands

**Figure 1:**
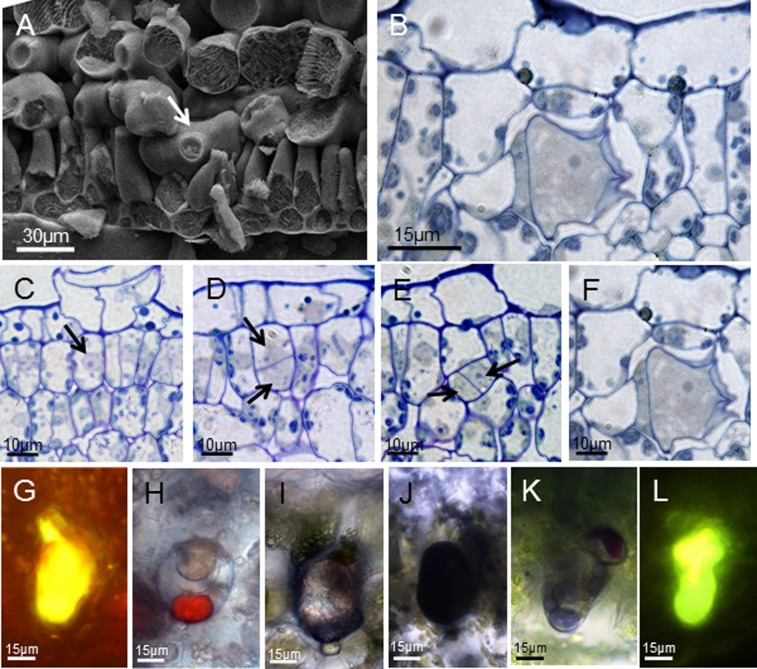
The development and histochemistry of internal glands in leaves. (A)The images (SEM) of *Pogostemon cablin* leaves showing the internal gland (arrow) among palisade cells. (B) The semithin section of leaves showing the morphology of internal gland. (C–F) Semithin sections of internal glands in different developmental phases showing the developmental process: (C) the initial cell of internal glands with the nucleus (arrow); (D) the initial cell with a vacuolate basal cell and a apical cell after apericlinal cell division (arrows); (E) internal glands at three-celled stage with twocytoplasmically dense cells after the apical cell divisions (arrows); (F) mature internal glands with one big secretory cell, one narrow stalk cell and one vacuolate basal cell. (G–L) Bright field and fluorescence micrographs of internal glands in leaves showing histochemical characterization of secretory products. Secretory material reacts positively for total lipids with Neutral Red (G), Sudan III (H) and Sudan Black B (I). The reaction for unsaturated lipids using OsO_4_ (J) is positive. The essential oil within the sub-cuticular space has reacted positively with the Nadi reagent for terpenes (K). And the staining for flavones with Naturstoffreagent A suggests the presence of flavones in the internal glands of leaves (L).

**Figure 2:**
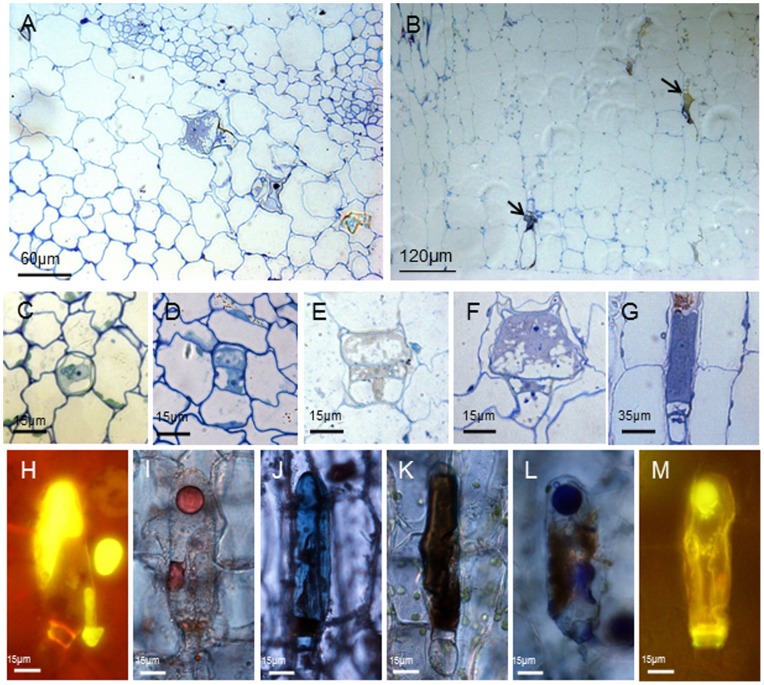
The development and histochemistry of internal glands in stems. Transverse (A) or longitudinal (B) section of stems showing the internal glands (arrows) among the cortical cells. (C–G) Light micrographs of internal glands in different developmental phases showing the process of development: (C) the initial cell with nucleus and few vacuoles; (D) two-celled stage with two cytoplasmically dense cells after cell divisions; (E) three-celled stage with one vacuolate basal cell, one narrow stalk cell, and the apical initial cell; (F) pre-secretory stage with one big apical cell; (G) mature internal glands with a long secretory cell. (H–M) Bright field and fluorescence micrographs of internal glands in stems showing histochemical characterization of secretory products. The staining for total lipids with Neutral red (H), Sudan III (I) and Sudan Black B (J) suggests the accumulation of total lipids in the internal glands of stems. In addition, the presence of unsaturated lipids is demonstrated by the reaction with OsO_4_ (K). NADI staining for terpenes is positive in mature internal glands (L). Secreted material in the sub-cuticular space of internal glands reacts positively with Naturstoffreagent A for the detection of flavones (M).

The adaxial and abaxial surfaces of the investigated leaves and stems of *Pogostemon cablin* showed numerous glandular trichomes and bristle hairs (Figure S1). According to the morphology of glandular trichomes, three types of trichomes were observed. Each of the three external trichome types of *Pogostemon cablin* could be easily characterized. Two of three external trichomes were short-stalked capitate (type I) and peltate trichome (type II) and one was long-stalked capitate trichome (type III) in *Pogostemon cablin*. Internal glands were found in the palisade tissue of leaves (type IV) (Figure 1A and B) and in the cortex parenchyma of stems (type V) (Figure 2A and B). Every external trichome type started its development with an epidermal cell, which first underwent a periclinal cell division (Figure S3). Various colour reactions (Figure S4) indicated that the secretion stored in the sub-cuticular space (SCS) of mature external trichomes contained hydrophilic and lipophilic components (Table 1). The head regions in secretory external trichomes of *Pogostemon cablin* differed in size, structure, composition of the metabolites and the secretory process.

**Table 1:** Histochemistry of the mature secretory glands of *Pogostemon cablin*.

Staining procedure	Target compounds	Colour observed	Type I	Type II	Type III	Type IV	Type V
Ruthenium red	Mucilage/pectin	Red to pink	+	+	−	−	−
Neutral red	Total lipids	Red	+	+	+	+	+
Sudan III	Total lipids	Orange	+	+	+	+	+
Osmium tetroxide	Unsaturated lipids	Brownish to black	+	+	+	+	+
Sudan black B	Total lipids	Dark blue to black	+	+	+	+	+
Naturstoffreagent A	Flavonoids	Yellow to orange	+	−	−	+	+
PAS	Neutral polysaccharides	Red	+	+	−	−	−
NADI reagent	Terpenes	Violet-blue	+	+	+	+	+

−, negative; +, positive.

**Figure 4:**
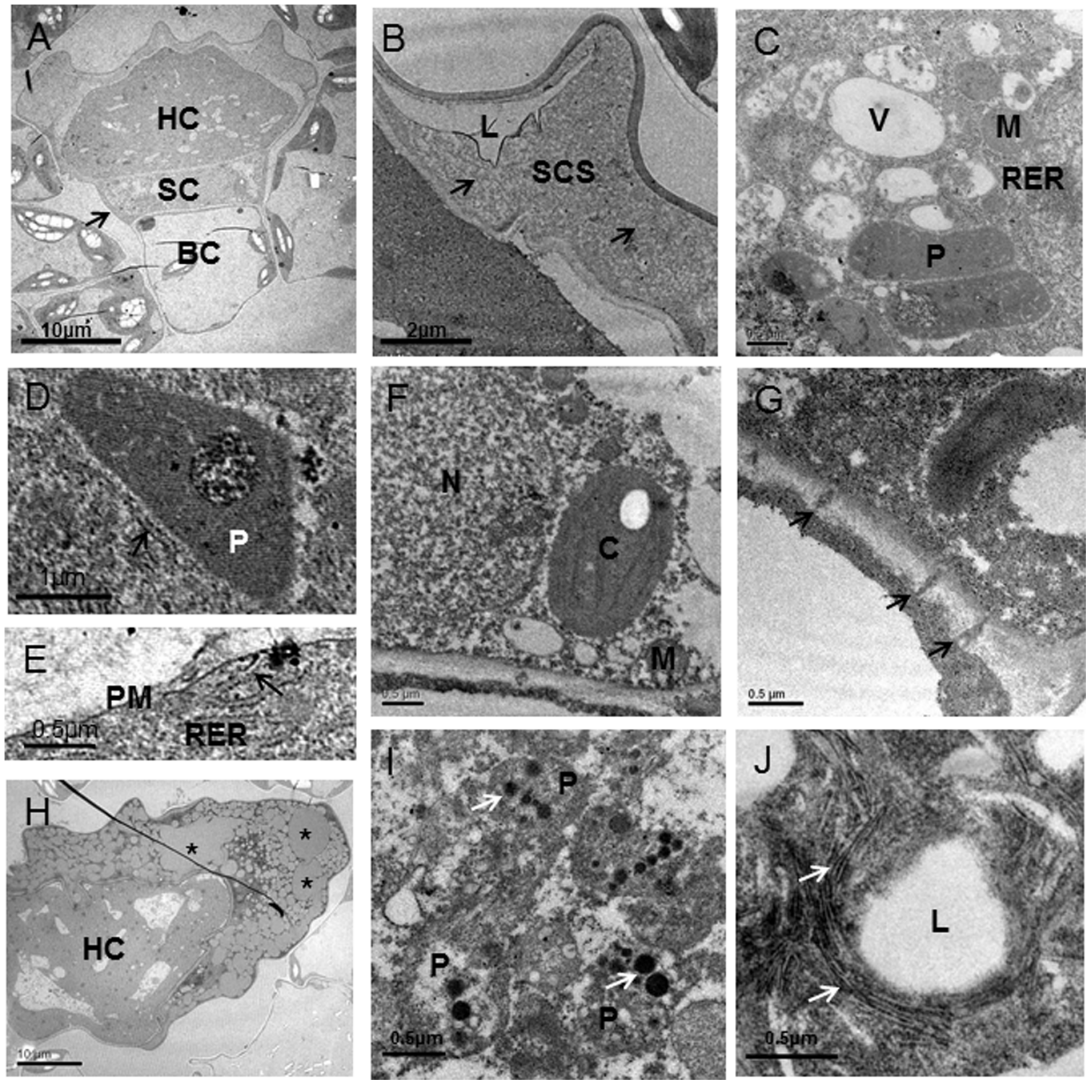
Ultrastructural aspects of internal glands in leaves of *Pogostemon cablin*. An internal gland at secretory stage shows the unique characteristics (A–G). (A) A mature internal glands has a cytoplasmically dense head cell (HC), a narrow stalk cell (SC) with densely stained lateral cell wall (arrow) and a basal cell (BC). (B) The sub-cuticular space (SCS) contains fibrillar material (arrows) and lipid-like material (L). (C) There are numerous small vacuoles (V), mitochondria (M), abundant rough endoplasmic reticulum (RER) and many plastids (P) in the head cell. (D) The plastids (P) are observed to be in close to RER (arrow). (E) And the RER (arrow) is also close to the plasma membrane (PM). (F) The details of the stalk cell show the big nucleus (N), the plump chloroplast (C) and numerous mitochondria (M). (G) A part of transverse wall between stalk cell and head cell is penetrated by numerous plasmodesmata (arrows). (H–J) The ultrastructural aspects of internal glands at late-secretory stage are observed as follows. (H) The sub-cuticular space is filled with numerous lipid spherosomes (_*_). (I) Plastids (P) contain numerous oil droplets (arrows). (J) Lipid droplets (L) in sub-cuticular space are surrounded by silk-like structure (arrows).

Internal glands in leaves with a length of 40 µm (±9) distributed among palisade cells (Figure 1A, arrow). Mature internal glands with a basal cell, a narrow stalk cell and one big cytoplasmically dense head cell had a SCS which was often filled with secretory oil (Figure 1B and Figure 4H).

The internal glands in leaves originated from a single undifferentiated palisade cell-like which had a larger nucleus and nucleolus (Figure 1C, arrow). The initial cell of internal glands formed one big vacuolated basal cell and one small, cytoplasmically dense apical cell through the unequal periclinal division (Figure 1D, arrows). As the development processed, the apical cell formed one narrow stalk cell and one big head cell with nucleus and nucleolus (Figure 1E, arrows). Afterward, the head cell enlarged to form the secretory cell which had a dense cytoplasm and large nuclei with prominent nucleoli (Figure 1F). When the internal glands came into secretory stage, the composition and the localization of the secretory compounds in SCS could be detected. The histochemical tests for detecting lipids compounds have given the positive results in this internal gland. Gold-yellow staining of secretion in the head and ‘neck-cell’ area with Neutral red under UV light indicated the presence of total lipids (Figure 1G), although staining with Sudan black B for total lipids was slightly positive (Figure 1I). Staining with Sudan III showed up faintly in the head cell, but the oil droplet contained in the SCS showed an intense jacinth staining (Figure 1H). These results were also confirmed using OsO_4_ for unsaturated lipids (Figure 1J). The Nadi reaction showed up an intense violet or blue-violet staining of the oil droplet contained in the SCS (Figure 1K). The head cell of mature internal gland became stained fluorescent yellow-orange with the Naturstoffreagent A which indicated flavones (Figure 1L). The histochemical tests carried out to detect polysaccharides compound, using ruthenium Red and PAS, showed up very weak reactions (not shown).

Another internal glands type with a length of 120 µm (±25) distributed among cortex cells of stems (Figure 2A and B). The internal glands, differing from external glandular trichomes or internal glands in leaves, originated from a signal meristematic cell close to phloem with nucleus and nucleolus (Figure 2C). Through a series of anticlinal division, a uniseriate row of three cells was formed (Figure 2D and E). The lower cell of the row corresponded to the basal cell of glands, the upper to the glands head and the intermediate to the stalk.

In the young internal gland of stems, the head cell did not have cuticle outer cell wall and was much smaller than that of mature internal glands in size (Figure 2E). With a further thickening of stem, the head cell lengthened and was covered with a thick cuticle. Fully-developed internal glands consisted of one long, cytoplasmically dense and cuticle-covered head cell, one narrow stalk cell and one vacuolated basal cell (Figure 2G). When subjected to UV light, the secretory material staining with Neutral red contained in the SCS revealed an intense gold-yellow secondary fluorescence, whereas slight chloroplasts fluorescing red (chlorophyll autofluorescence) were seen in stalk region (Figure 2H). The material secreted into the SCS contained lipophilic substances as tested with Sudan III (Figure 2I) and Sudan black B (Figure 2J). Staining with OsO_4_ (Figure 2K), for unsaturated lipids, showed positive results which confirmed the results of the tests using Neutral red, Sudan III and Sudan black B. The Nadi reaction resulting in an intense violet or blue-violet staining of the secretion contained in the SCS indicated the presence of terpenoids (Figure 2L). The fluorochrome for flavonoid detection, Naturstoffreagenz A induced a yellow-green secondary fluorescence in head cell and secreted exudate (Figure 2M). Similar to internal glands of leaves, the glands in stems gave negative or weak reactions with the tests used for polysaccharides compound.

The release of secreted exudate in SCS of the two internal gland types to the environment through cuticle was not observed. However, each of the three external trichome types of *Pogostemon cablin* showed intense secretion release in different ways under cryo-SEM (Figure S2). The secreted material was possibly released via the random fibrillar network on the reticulate cuticle of short-stalked capitate glandular trichomes or the gaps between the spherical accumulations of cutin at the apex of peltate glandular trichomes (Figure S5). The cuticle of external and internal glands showed different thicknesses, whereas the cuticle of internal glands of leaves had similar morphology and thicknesses with that of internal glands of stems (Figure S5).

### Ultrastructure of External Glands

**Figure 3:**
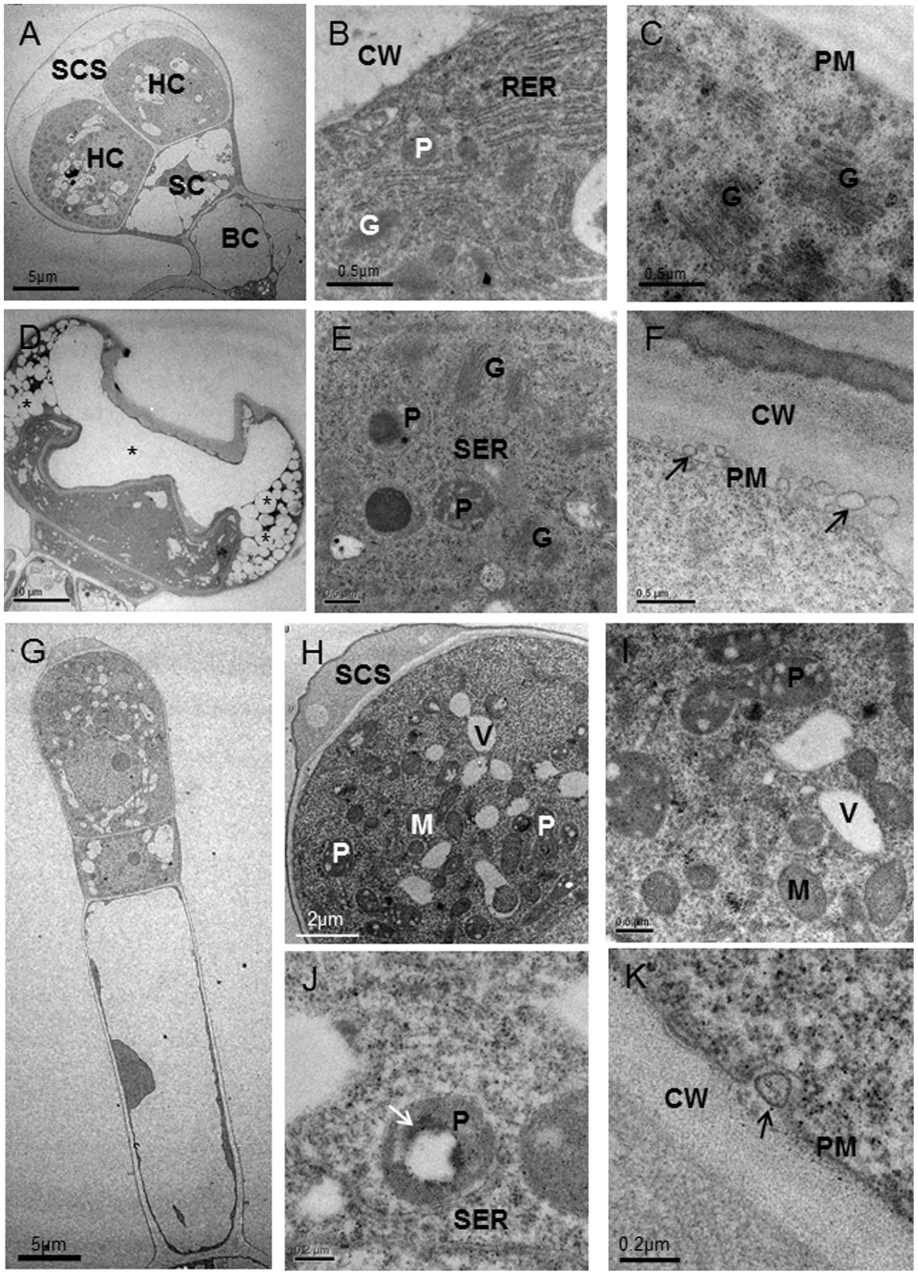
Ultrastructural aspects of three glandular trichome types of *Pogostemon cablin*. (A–C) Short-stalked capitate trichomes in secretory stage: (A) the glandular trichomes with two head cells (HC), a narrow stalk cell (SC) and a basal cell (BC) and the sub-cuticular space (SCS); (B) portion of head cell showing Golgi (G) and plastids (P) in close contact with rough endoplasmic reticulum (RER) near cell wall (CW); (C) numerous Golgi (G) with many vesicles near the plasma membrane (PM). (D–F) Peltate glandular trichomes in secretory stage: (D) longitudinal section through a secretory peltate glandular trichome with plentiful electron-light lipid deposits (_*_) in the sub-cuticular space; (E) the higher magnification of (D) showing Golgi (G) and plastids (P) in close contact with the short segments of smooth endoplasmic reticulum (SER); (F) vesicles (arrows) are found between cell wall (CW) and plasma membrane (PM). (G–K) Ultrastructural aspects of the long-stalked capitate trichomes: (G) the mature glandular trichomes with a cytoplasmically dense apical cell, a narrow stalk cell and an elongated vacuolated stalk cell; (H) the higher magnification of (G) showing the secretory cell with the sub-cuticular space (SCS), many small vacuoles (V), abandent mitochondria (M) and numerous small plastids (P); (I) the details of the cytoplasm of the secretory cell showing prevalence of mitochondria (M) and plastids (P) with plastoglobuli in the apical region; (J) the sparse smooth endoplasmic reticulum (SER) close to a lipid-filled plastid (P) (arrow); (K) a bigger vesicle (arrow) between cell wall (CW) and plasma membrane (PM).

In the secretory stage, the head cells of three external trichome types of *Pogostemon cablin* showed different ultrastructural features, although they all had dense cytoplasm with nucleus and nucleolus, numerous plastids and small vacuole. In the two secretory cells of short-stalked capitate glandular trichomes, long and narrow cisternae of rough endoplasmic reticulum, forming stacks, were found in the parietal cytoplasm, lying parallel to each other and to the plasma membrane (Figure 3A and B). And numerous Golgi vesicles were found in head cell and close to plasma membrane (Figure 3C). Unlike short-stalked capitate trichomes, the secretory cell of peltate glandular trichomes contained numerous short segments of SER which surrounded Golgi and plastids (Figure 3D and E). And many vesicles between plasma membrane and cell wall were observed (Figure 3F, arrows). In contrast to short-stalked capitate and peltate glandular trichomes, the long-stalked capitate glandular trichomes contained fewer long SER and sparse Golgi (Figure 3G and J). And the apical cell was strongly polarized. Numerous small vacuoles occupied the middle and basal parts of the cell and surrounded the nucleus (Figure 3G and H). The plastidome consisted of amoeboid plastids that often contained moderately large plastoglobuli and lipid-like material and occupied the apical region of the secretory cell (Figure 3I and J). Similar to peltate glandular trichomes, one vesicle between cell wall and plasma membrane was present in several specimens (Figure 3K).

### Ultrastructure of Internal Glands

The internal glands located in mesophyll parenchyma ultrastructurally resembled peltate trichomes (Figure 4A). In early secretory stage, the head cell of internal glands was characterized by enlarged plastids, numerous small vacuoles, an extensive short rough endoplasmic reticulum (RER) and the detachment of the thick cuticle from the outer cell walls to form an extensive extracellular SCS (Figure 4B and C). In SCS, a smooth-textured, lipid-like material occurred within the fibrillar substance as thin, sheet-like layers (Figure 4B). The mature plastids containing small plastoglobuli were surrounded by short RER segments (Figure 4C). RER appeared to closely approach or contact plastids and osmiophilic deposits were consistently present along RER segments near the plastids (Figure 4C and D). In addition, RER seemed to be in close contact with plasma membrane, indicating the continuity between RER and plasma membrance (Figure 4E). In the late secretory stage, SCS filled with numerous lipid spherosomes surrounded by a lot of fibrillar substance became very big (Figure 4J). Plastids often contained large plastoglobuli and oil droplets (Figure 4I). Unlike capitate trichomes, the internal glands lacked Golgi bodies and large vesicles but, numerous mitochondria were found. The lateral cell wall of the narrow stalk cell thickened and had become densely staining (Figure 4A, arrow). The narrow stalk cell with dense cytoplasm contained larger nucleus and elliptical chloroplasts with starch grains. Abandent small mitochondria and a few of small vacuoles were also observed in the narrow stalk cell (Figure 4F). The basal cell with natural chloroplasts remained vacuolate, and its peripheral cytoplasm appeared to contain fewer organelles than the stalk or secretory cell (Figure 4A). The periclinal cell wall, bordering the stalk cell, usually contained branched plasmodesmata (Figure 4G).

**Figure 5:**
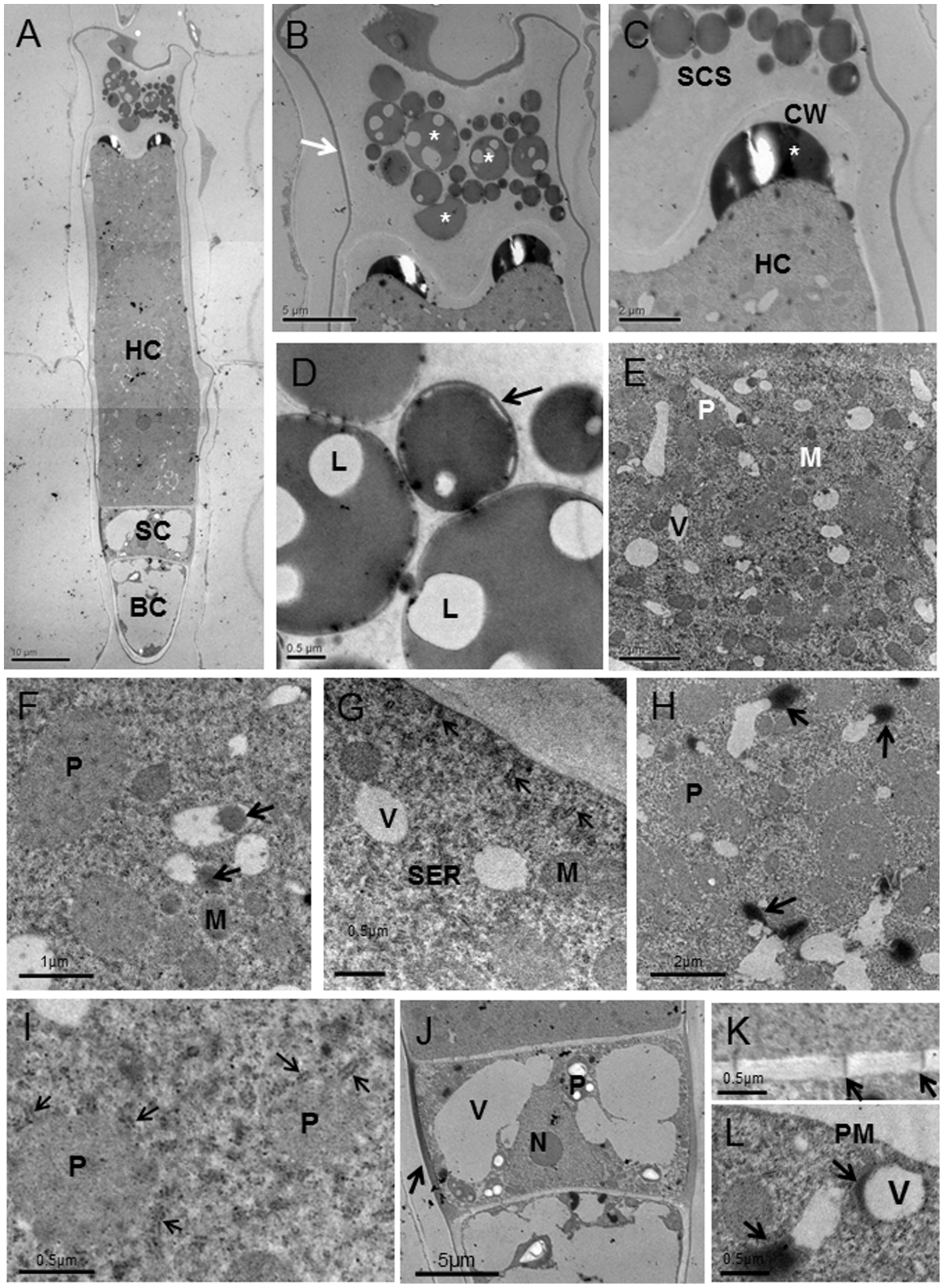
Ultrastructural aspects of internal glands in stems of *Pogostemon cablin*. (A) Longitudinal section of internal glands showing that the internal gland has a long head cell (HC), a stalk cell (SC) and a basal cell (BC). (B) The sub-cuticular space (SCS) of internal glands has thin cuticle (arrow) and is filled with oil droplets (_*_). (C) The higher magnification of (B) showing one big oil droplet (_*_) between cell wall (CW) and plasma membrane. (D) Oil droplets in SCS contain lipid droplets (L) and membrane-like structure (arrow) outside the oil droplet. (E) Portion of head cell showing numerous mitochondria (M), small vacuoles (V) and plastids (P). (F) Vacuoles with electron-opaque material (arrows) are in close to plastid (P) and mitochondria (M). (G) The smooth endoplasmic reticulum (arrows) is observed to be in close to plasma membrane (PM). (H) Portion of mature internal glands showing electron-opaque material (arrows) in close contact with vacuoles. (I) The smooth endoplasmic reticulum (arrows) in close to plastids (P). (J) The stalk cell with thickened lateral wall (arrow) contains big vacuoles (V), the nucleus (N) and numerous plastids (P). (K) The details of cell wall showing plasmodesmata (arrows) that connect the narrow stalk cell and head cell. (L) The details of head cell showing that vesicles (V) near plasma membrane (PM) are in close connect with electron-opaque material (arrows).

Compared with internal glands in leaves, the internal glands of stems had one longer, cytoplasmically dense and cuticle-covered head cell, one narrow stalk cell and one vacuolated basal cell (Figure 5A). In secretory stage, the SCS formed by the detachment of the thick cuticle from the outer cell walls was filled with numerous spherosomes (Figure 5B, asterisks). The secreted material, stored in the periplasmic space (Figure 5C, asterisks), passing through the wall, accumulated temporarily in the SCS. The oil droplets surrounded by membrane-like structure (arrow) in SCS contained lipid droplets and electron-opaque material outside the oil droplets (Figure 5D). After the head cell reached the characteristic structure of a fully-developed gland, numerous mature plastids with small plastoglobuli, the marked proliferation of the endomembrane system, an increase in the number of mitochondria and a lot of small vacuoles were the remarkable feature of the secretory cell (Figure 5E and I). In this stage, the secretory cell contained two nuclei with clear nucleolus (Figure 5A). Plastids were found only in secretory cells, usually in close contact with short cisternae of smooth endoplasmic reticulum (SER) (Figure 5I, arrows). These plastids were variable in form and lacked chloroplasts and starch grains (Figure 5F and I). The small vacuoles near plastids and short cisternae of SER often contained larger highly osmiophilic spherosomes (Figure 5F). At the apical region of the secretory cell, vacuoles variable in form seemed to be exhausted and in close contact with electron-opaque material (Figure 5H and L). In addition, vacuoles with electron-opaque material were often observed near the plasma membrane (Figure 5L). Short segments of SER filled with osmiophilic droplets appeared closely approach or contacted the plasma membrane and plastids (Figure 5G and I). Numerous mitochondria were extensively distributed within the cytoplasm near other organelles (Figure 5E). The Golgi was sparse and not well-developed. The stalk cell had two big vacuoles and cellulosic cell walls that were stained black. The cytoplasm was dense and had several mitochondria. The stalk cell contained one triangular nucleus with clear nucleolus and many plastids with big stanch grains (Figure 5J). The basal cell was highly vacuolated and its peripheral cytoplasm appeared to contain fewer organelles than the stalk or secretory cells. Plasmodesmata occurred frequently between the head cell and the stalk cell, and also occurred between the basal cell and the stalk cell, but were less numerous (Figure 5K). There was not obvious connection between the internal gland and parenchymal cells around.

## Discussion

### Development and Histochemistry

Observations on transverse section of leaves showed internal glands of *Pogostemon cablin* distributed among palisade cells, which was similar to the intact sub-dermal secretory cavities of *Eucalyptus* [21] and elaiophores of *Oncidium trulliferum* [22]. In contrast to the elaiophores of *Oncidium trulliferum* which secreted and stored oils under a distinct subepithelial layer, the oils of the internal glands were secreted and stored in the SCS like the external glandular trichomes. Compared to the internal glands in leaves, numerous internal glands with long head cell among cortex cells of stems had bigger SCS filled with oil droplets. Observations on the development of the two internal glands types showed that the glands of stems originated from a single meristematic cell close to phloem and the internal glands in leaves originated from one undifferentiated palisade cell. These results indicated that the two internal gland types both originated from similar ground meristem. The absence of the two internal gland types in primordial leaves and apical dome of the stem apex suggested cellular differentiation at a later stage during the maturation of stems and leaves. However, cell differentiation terminates in early leaf development, which is described by Cutter [23]. These observations may not provide further evidence for this hypothesis.

An abundance of terpenoids was reported in the leaves and stems of *Pogostemon cablin* [18]. Monoterpenes dominate the secretory material of Rutaceae species [24] and the Lamiaceae [25]; Sesquiterpenes and other terpenes are the main components of the essential oil of the Asteraceae [26]. Terpenoids have many different functions in plants such as attracting pollinating insects to flowers or protecting the plant from destruction by herbivores and other pathogens [27], [28]. The result of the Nadi reagent test revealed the presence of the terpenoids in the secretory material stored in SCS of all mature gland types of *Pogostemon cablin*. The abundant terpenoids in the external and internal glands of *Pogostemon cablin* may protect the plant from destruction by herbivores through influencing their alimentary system. Histochemical test on the two fully developed internal glands indicated that the secretion contained lipophilic components. These results are similar to the phytochemical data available for other species of the Lamiaceae [29]. The presence of lipid compounds in palisade tissue has also been reported by Maria et al. [30] in *Lantana camara*. Only the internal glands and short-stalked capitate glandular trichomes contained flavones. The flavones had a diverse array of physiological functions, not only acting as antioxidants and/or sunscreen pigments to protect plants from oxidative and UV light damage but also as modulators of auxin transport [31], [32]. These functions of flavones had been accentuated by the presence of flavonoids in internal glands. And polysaccharides were found only in the two capitate glandular trichome types. The different composition of the secretory material in the five secretory gland types may suggest that the function of these epidermal appendages and internal glands differed from each other.

Interestingly, the composition of the secretory material in internal glands which distributed among the cortical cells of stems was similar with the internal glands in leaves. And the two internal gland types with similar internal cuticle both originated from ground meristem. Taking the similarities in development, morphology and histochemistry between the two internal gland types into consideration, it is possible that these two secretory gland types have close evolutionary relationship, although their locations and size are different. And the similar structures of two internal gland types with peltate glandular trichomes, including a sub-cuticular space filled with secretory material and three components: a basal region, a stalk region and a head region, lead us to purpose that the internal glands of *Pogostemon cablin* may be the similar secretory structure type with peltate glandular trichomes. However, the internal glands had different origin compared with external glandular trichomes. Possible differences within the secretory glands, as observed through developmental differences between internal glands and external glandular trichomes, were accentuated by the absence of polysaccharides and the presence of flavonoids in internal glands. In addition, the distribution of glands in *Pogostemon cablin* and the absence of secretion release in internal glands also indicated the difference between internal glands and external glands.

The stalk cells of the mature internal glands observed in this study all appeared to contain densely-stained cytoplasm, unusual plastids with starch grains, and an intact nucleus, occasionally with a moderately large nucleolus. These observations, together with the presence of obvious plasmodesmata on the periclinal walls of stalk cells and the lack of chloroplasts in the head cells indicate that it is likely that these cells are related to their essential role in the supply of carbon substrates to the non-photosynthetic head cells. In addition, the lateral wall of the stalk cells of external glandular trichomes forms a boundary to the exterior and is heavily suberized. It is interesting that similar suberization of lateral wall was observed in internal glands. The thickened lateral wall is deemed to contribute to support the head cell, as supposed by previous work. As the internal glands are among the palisade cells or cortex cells, it is unlikely that stalk cells are specialized for supporting the head cell. The result of histochemistry tests of mature internal glands showed that there was weak reaction in stalk cells. It seems that at least some of the specializations of stalk cells of internal glands are related to essential oil biosynthesis, not only supply of carbon substrates.

### Site of Oil Biosynthesis

In the previous studies about secretory cells of external glands from taxonomically distant plants, similar ultrastructural features, including dense cytoplasm, extensive endoplasmic reticulum, amoeboid leucoplasts, relatively few Golgi, and abundant mitochondria, have been noted [13], [16], [33]–[34]. Basing largely upon observations of the apparent sites of lipid accumulation, previous investigators supposed the possible sites of oil biosynthesis such as SER [35], vacuoles [33], cytoplasm [36], and the combination of plastids and SER [37]. However, there is a lack of information on the site of monoterpene and lipids biosynthesis of the internal glands in *Pogostemon cablin*.

The observations obtained by TEM showed that the internal glands of leaves in early secretory stage contained abundant small plastids which were reported to be found only in external glandular trchomes. Following the accumulation of secretory oil in the SCS, the plastids became mature and contained internal membranes and small plastoglobuli, similar to that noted in other plants by Glenn et al. [11] and Franceschi and Giaquinta [38]. The oil droplets in mature plastids strongly indicated that the plastids may play an important role in oil biosynthesis. The osmiophilic inclusions-containing RER and the very close association with plastids suggests an important role for RER in lipids biosynthesis, as suggested in *Peppermint* by Glenn and Rodney [39].

Similar to the internal glands of leaves, the main ultrastructural characteristics of the fully secreting internal glands in stems, including numerous plastids in close contact with a well-developed tubular membrane system filled with osmiophilic inclusions, abundant mitochondria, small vacuoles containing electron-opaque material, poorly developed Golgi bodies and lipid-like material in close contact with small vacuole, were first observed in the present work. These observations supply evidence that internal glands in stems are typical lipid and monoterpenes secreting glands. Although the lipids-containing plastids were not observed in internal glands of stems, the extensive development of the plastid compartment with profuse tubular osmiophilic structures, together with the high proliferation of the SER filled with osmiophilic inclusions, indicated that the plastids and SER may play an important role in lipids and monoterpenes biosynthesis, as suggested in other plants [11], [16], [37]. Amelunxen [33] has supposed that vacuoles are the possible sites of terpenoid synthesis. In the long secretory cell of internal glands in stems of *Pogostemon cablin*, small vacuoles containing electron-opaque material and lipid-like material in close contact with small vacuoles strongly suggested that the vacuoles may have an important role in lipids biosynthesis. In addition, during the excretion of the lipid-like material out of small vacuoles, the vacuoles seemed to be exhausted anomalous and the extraplasmatic space was much enlarged. Thus, the possible role of vacuoles in lipids biosynthesis has been accentuated by these observations. However, no futher evidence has been ovserved to support the hypothesis. As supposed by previous researchers [6], [34], [40], vacuoles may not produce, but only process the secretory material.

Histochemical staining has shown that, in addition to lipids and terpenoids, the internal glands in stems and leaves of *Pogostemon cablin* also contained abundant flavones. And it is possible that some of the ultrastructural features described here may represent specializations for the biosynthesis of the flavones. The remarkable changes of SER during the development of internal glands may indicate the role of SER in flavones biosynthesis, which has been suggested by the location of flavonoid and phenylpropanoid biosynthetic enzymes in ER [41], [42].

Similar to internal glands, plastids in three external glandular trichome types of *Pogostemon cablin* were the organelles that showed the most striking changes during the development of the trichomes. Although the secretory cells of peltate trichomes and short-stalked capitate trichomes in this work did not contain the lipid-containing plastids which could indicate the important role of plastids in the oil biosynthesis, the correlation between the striking changes of plastids and the secretory process may suggest that the plastids had a role in lipids and monoterpene biosynthesis. And the lipid-containing plastids in long-stalked capitate trichomes strongly indicated the important role of plastids in the lipids biosynthesis. However, the difference in the number of ER and Golgi among all the glands types of *Pogostemon cablin* may suggest their different roles in the oil biosynthesis.

### Possible Secretory Mechanisms

Basing upon the very close association of SER with plastids and the plasma membrane, and the presence of lipid deposits within the SER, previous investigators postulated a secretion mechanism for secretory material mediated by direct SER-plasma membrane connections [43]–[45]. Multivesicular bodies, as noted by Tanchak and Fowke [46] and Tse et al. [47], connect with both endocytosis and secretion. And Paramural bodies, as reported in *Claceolaria* trichomes [48] and *Genlisea* Digestive Hairs [49], may also play an important role in the transport of synthesized material.

In the internal glands of leaves and stems of *Pogostemon cablin*, we suggest that the secretory material may also be transported directly from ER to the cell wall via connecting ER membranes with the plasmalemma. The abundent secretory oil in the peripheral cytoplasm and periplasmic space, as described in *Zeyheria Montana* by Silvia et al. [16], indicated the accumulation of oil not through multivesicular bodies or paramural bodies. Many previous investigators have noted similar ultrastructural features of the secretory oil in the SCS of mature external glands. Unlike the electron-light lipid deposits in other secretory glands of *Pogostemon cablin*, the secretory material in the SCS of mature internal glands in stems appeared many spherosomes with membrane-like structure and electron-light lipid droplets among electron-opaque substance. We suppose that the electron-opaque substance in SCS is as same as the electron-opaque material in close contact with small vacuoles. And the small vacuole with electron-opaque material close to plasma membrane suggested that the export of this material from the secretory cells to the storage space may be similar to the lanthanum transport mechanisms in *Atriplex halimus* [50], although a series of oil-containing small vacuole connecting the big vacuole and the plasma membrane were not observed. And the SCS of internal glands in leaves filled with numerous lipid spherosomes surrounded by a lot of fibrillar substance whose function was not clear.

However, not only RER in close contact with plasma membrane was observed, but also abundant vesicles which were similar to paramural bodies were observed in the secretory cells of peltate glandular trichomes. In addition, abundant Golgi stacks with numerous bodies of the short-stalked capitate trichomes during the secretory phase were observed to have close approach with RER and plasma membrane. In the secretory cell of long-stalked capitate trichomes, the vesicle was observed between cell wall and plasma membrane. Thus, it is possible that there are several ways in which secretory material is transported in external glands. We suggest a similar secretion mechanism to internal glands for short-stalked capitate trichomes by direct SER-plasma membrane connections. And abundant vesicles between cell wall and plasma membrane in peltate trichomes and long-stalked capitate trichomes may suggest the export of secretory oil by the paramural bodies process, as postulated in *Claceolaria* trichomes [48] and *Genlisea* Digestive Hairs [49]. The Golgi bodies in close contact with plasma membrane suggest that the final secretion product may be transported to the cell surface via Golgi vesicles, and released into the periplasmic space through the fusion of Golgi vesicles with plasma membrane. A similar mechanism of secretion has been reported in other trichomes [13], [51]. The way in which the secretory oil is transported from secretory cells to SCS may provide new evidence to point out the difference between the external and internal glands. And the different way of essential oil secretion in different glands may indicate the diversity of the export of substance.

In summary, the data on developmental stage, histochemistry and ultrastructures about the site of oil biosynthesis and secretory mechanism of internal glands in the study indicated that the internal glands may be a novel secretory structure distributed within the plant which was different from external glands on leaves, although they all had similar structures. The internal glands appeared inside stems and leaves and occurred later than external glands during the development that may reveal the adaptability of secretory glands to plant diseases and insect pests stress in long term evolution.

The identical composition of the secretory material and the similar ultrastructure between the two types of internal glands, including plastids with similar shape, poorly developed Golgi bodies, numerous short cisternaes of rough endoplasmic reticulum and thin cuticle, indicated that there may be close evolutionary relationship between the two internal gland types. And the distribution of internal glands from leaves to stems may also further reflect its biological function and adaptability to environment in different plant organs. Each of these lipid secreting glands had its specific ultrastructure which may reveal the site of lipid biosynthesis and may have different function. They demonstrated a different way of secretory processing and releasing. These results indicated the diversity of the site of oil biosynthesis and secretory mechanisms of secretory glands in a given species. For the question as to why and for what *Pogostemon cablin* leaves and stems need all these different gland types, further clarification of the functions and ecophysiological and evolutionary roles of these secretory glands is required.

## Materials and Methods

### Plant Material

To establish the experiments, *Pogostemon cablin* was grown in growth chambers under defined climatic conditions with a photoperiod of 16 h. Day and night temperatures were respectively 22°C and 18°C. And the relative humidity was from 50% to 70%. Fresh leaves at various developmental stages of maturity were selected for the investigation of glandular trichomes and internal glands. Young stem and mature stem were harvested for the study of internal glands.

### Scanning (SEM) and Transmission (TEM) Electron Microscopy

For conventional scanning electron microscopy (CSEM), leaves and stems at various developmental stages were fixed in glutaraldehyde (2.5% with 0.1 M phosphate buffer, at pH 7.3, overnight at 4°C). Sectional material was washed in the phosphate buffer (pH 7.3). Dehydration was done in an acetone dilution series (30%, 50%, 70%, 90%, followed by 3×100%). After critical drying with Leica EM CPD300 automated critical point dryer, the samples were mounted on double-sided carbon tape on stubs. They were then plasma coated with 10 nm gold and viewed with a Hitachi S-3400N scanning electron microscope. For cryo-SEM, samples were fixed in liquid nitrogen, sublimated and gold-coated in Quorum PP2000T Cryo-SEM system. For transmission electron microscopy, samples were fixed in glutaraldehyde (2.5% with 0.1 M phosphate buffer, at pH 7.3), post-fixed in osmium tetroxide (1%, in 0.1 M phosphate buffer, pH 7.3), dehydrated in an acetone dilution series and infiltrated with Eponate 12 resin. Sections for transmission electron microscopy were cut to a thickness of 70 nm with diamond knives and a Leica EM UC6 ultramicrotome. The sections were stained with either 1% (w/v) aqueous uranyl acetate and 1% (w/v) lead citrate. Specimens were viewed with a JEM-1230 (JEOL, Tokyo) transmission electron microscope.

### Light Microscopy

The development and histochemistry of external trichomes and internal glands were studied with light microscopy. Sections for light microscopy were cut to a thickness of 0.5 to 1 µm with glass knives and stained with toluidine blue. The main classes of metabolites in secreted material of glandular trichomes were observed in fresh and fixed hand-sections, using following different histochemical tests. Neutral red and Sudan black B were used to localize total lipids, osmium tetroxide for unsaturated lipids, Naturstoffreagent A for detection of flavonoids (under UV 365 emission LP 397), periodic acid-Schiff (PAS) reagent for polysaccharides, Sudan III for lipids, NADI reagent for terpenes, ruthenium red for pectins. The observations were made under an Olympus microscope.

## Supporting Information

Figure S1 **Morphology and distribution of glandular trichomes on the surface of** ***Pogostemon cablin.*** (A–F) (SEM): Adaxial surface (A) and abaxial surface (B) view with peltate glandular trichomes and short-stalked capitate glandular trichomes in secretory phase. (C) The surface view of stems showing long-stalked capitate trichomes (arrows). (D) Long-stalked capitate trichomes (arrows) among glandular trichomes and non-glandular trichomes on the leaf veins. (E) Distribution of glandular trichomes on stem apex of *Pogostemon cablin.* (F) Higher magnification of (E) showing fully developmental peltate glandular trichomes and short-stalked capitate glandular trichomes and the tuberculate epidermal cells depicted by arrows.
(TIF)

Figure S2 **SEM micrographs showing the morphology and the secretion of three glandular trichome types in** ***Pogostemon cablin*** **.** (A–C) The secretion of short-stalked capitate glandular trichomes: (A) Pre-secretory stage with the secretory centre (arrow); (B) beginning of the secretory release (arrow); (C) the secretory droplets are getting bigger and the number increase to two (arrows). (D–F) The secretion of long-stalked capitate glandular trichomes: (D) beginning of the secretory release and the oil droplet is small (arrow); (E) the oil droplet is getting bigger (arrow); (F) the small oil droplet (arrow) outside the apical cell. (G–I) The secretion of peltate glandular trichomes: (G) Pre-secretory stage without thickened cuticle; (H) secretory stage with protruding cuticle; (I) the collapse of the sub-cuticular space after secretion.
(TIF)

Figure S3 **Semithin sections of three glandular trichome types in different developmental phases showing the process of development.** (A–E) The development of short-stalked capitate glandular trichomes: (A) protruding epidermal cell with an asymmetrical cytoplasmic distribution containing vacuolate basal portions and cytoplasmically dense apical portions; (B) two-celled stage with one cytoplasmically dense apical cell and one vacuolate cell; (C) three-celled stage with one big cytoplasmically dense apical cell; (D) four-celled stage with two cytoplasmically dense apical cells without cuticle; (E) mature short-stalked capitate glandular trichomes with one sub-cuticular space containing essential oil (arrow). (F–J) The developmental process of long-stalked capitate glandular trichomes: (F) protruding epidermal cell with a vacuolate basal region and an apical region containing the nucleus; (G) glandular trichome initial after periclinal cell divisions with a vacuolate basal cell and a apical cell containing the nucleus in the apical region; (H) three-celled stage showing a vacuolate basal cell,a vacuolate stalk cell, and an apical region containing the nucleus; (I) glandular trichomes in pre-secretory stage with a cytoplasmically dense apical cell, a narrow stalk cell and an elongated stalk cell; (J) mature long-stalked glandular trichomes with one sub-cuticular space containing essential oil (arrow). (K–O) The developmental process of peltate glandular trichomes: (K) protruding epidermal cell with a vacuolate basal region and an apical region containing the nucleus; (L) two-celled stage with one cytoplasmically dense apical cell and one vacuolate basal cell; (M) three-celled stage with one cytoplasmically dense apical cell containing two nucleus, one narrow stalk cell and one vacuolate basal cell; (N) mature peltate glandular trichomes with one sub-cuticular space (arrow); (O) post-secretory glandular trichomes with the collapse of the sub-cuticular space.
(TIF)

Figure S4 **Bright field and fluorescence micrographs of three glandular trichome types showing histochemical characterization of secretory products.** (A–H) Histochemistry of the short-stalked capitate glandular trichomes: (A) Ruthenium Red test showing the apical cells stained red; (B) gold-yellow secondary fluorescence observed with Neutral Red under UV light; (C) positive staining reaction with Sudan III in the apical cells and weak reaction in the stalk cell; (D) the apical cells stained blue with Sudan Black B and the stalk cell stained black; (E) OsO_4_ test showing the apical cells and the droplet (arrow) stained black; (F) NADI staining for terpenes is positive in the apical cells; (G) the apical cells react positively with Naturstoffreagent A; (H) PAS test for polysaccharides in apical cells. (I–O) Histochemistry of the long-stalked capitate glandular trichomes: (I) ruthenium Red test showing the apical cells stained red; (J) yellow staining of secretion in sub-cuticular space with Neutral red; (K) secretory material stained with Sudan III; (L) positive staining reaction with Sudan Black B; (M) black staining of secretion with OsO_4_, secretory process is visible; (N) secretory material reacts positively for terpenes with NADI; (O) mature trichome reacts positively in PAS test for polysaccharides in the head cell and stalk cell. (P–T) Histochemistry of the peltate glandular trichomes: (P) gold-yellow secondary fluorescence observed with Neutral Red under UV light; (Q) secretory material in the sub-cuticular space positive stained with Sudan III; (R) staining for total lipids with Sudan Black B; (S) positive staining reaction with OsO_4_ in the head cells and weak reaction in the narrow stalk cell; (T) NADI staining for terpenes is positive in the sub-cuticular space.
(TIF)

Figure S5 **EM micrographs showing the cuticle of mature external and internal glands in** ***Pogostemon cablin*** **.** (A) A random fibrillar network (arrow) on the reticulate cuticle of short-stalked capitate glandular trichomes is evident; (B) the thin cuticle of long-stalked capitate glandular trichomes; (C) TEM and (D) SEM micrographs showing the gaps (arrow) between the spherical accumulations of cutin at the apex of peltate glandular trichomes; (E) the cuticle of internal glands in leaves with high electron density; (F) the cuticle of internal glands in stems.
(TIF)
