# Supplementary figures and images for: Development and Structure of Internal Glands and External Glandular Trichomes in Pogostemon cablin
Source: PLoS One. 2013 Oct 30;8(10):e77862. doi: 10.1371/journal.pone.0077862 (PMC3813755; doi:10.1371/journal.pone.0077862)

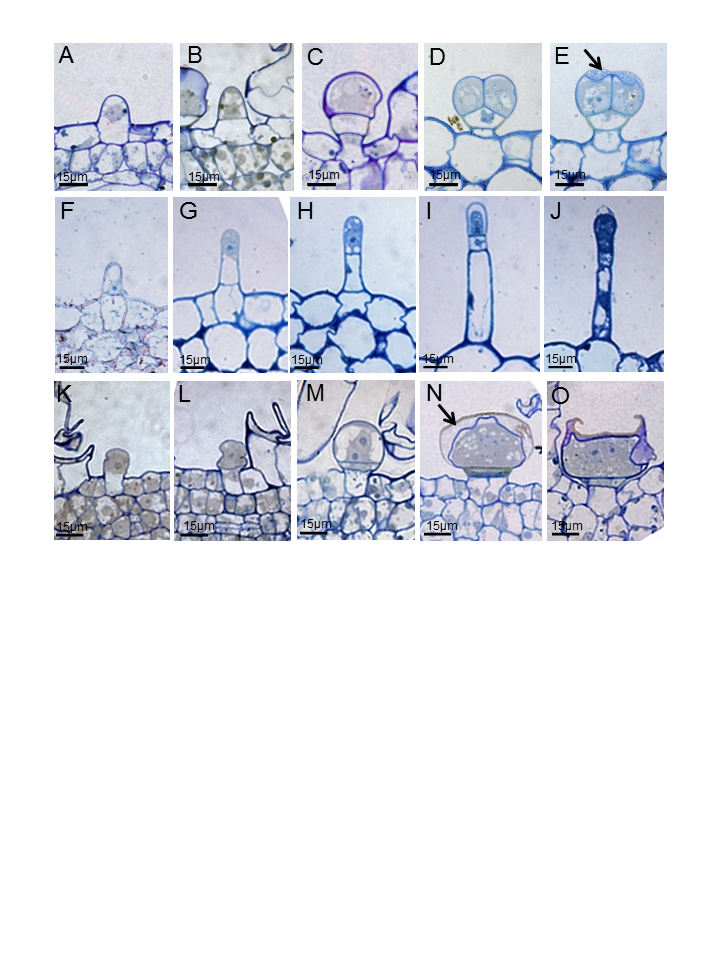

Supplement: Figure S3 — Semithin sections of three glandular trichome types in different developmental phases showing the process of development. (A–E) The development of short-stalked capitate glandular trichomes: (A) protruding epidermal cell with an asymmetrical cytoplasmic distribution containing vacuolate basal portions and cytoplasmically dense apical portions; (B) two-celled stage with one cytoplasmically dense apical cell and one vacuolate cell; (C) three-celled stage with one big cytoplasmically dense apical cell; (D) four-celled stage with two cytoplasmically dense apical cells without cuticle; (E) mature short-stalked capitate glandular trichomes with one sub-cuticular space containing essential oil (arrow). (F–J) The developmental process of long-stalked capitate glandular trichomes: (F) protruding epidermal cell with a vacuolate basal region and an apical region containing the nucleus; (G) glandular trichome initial after periclinal cell divisions with a vacuolate basal cell and a apical cell containing the nucleus in the apical region; (H) three-celled stage showing a vacuolate basal cell,a vacuolate stalk cell, and an apical region containing the nucleus; (I) glandular trichomes in pre-secretory stage with a cytoplasmically dense apical cell, a narrow stalk cell and an elongated stalk cell; (J) mature long-stalked glandular trichomes with one sub-cuticular space containing essential oil (arrow). (K–O) The developmental process of peltate glandular trichomes: (K) protruding epidermal cell with a vacuolate basal region and an apical region containing the nucleus; (L) two-celled stage with one cytoplasmically dense apical cell and one vacuolate basal cell; (M) three-celled stage with one cytoplasmically dense apical cell containing two nucleus, one narrow stalk cell and one vacuolate basal cell; (N) mature peltate glandular trichomes with one sub-cuticular space (arrow); (O) post-secretory glandular trichomes with the collapse of the sub-cuticular space. (TIF) [file pone.0077862.s003.tif]

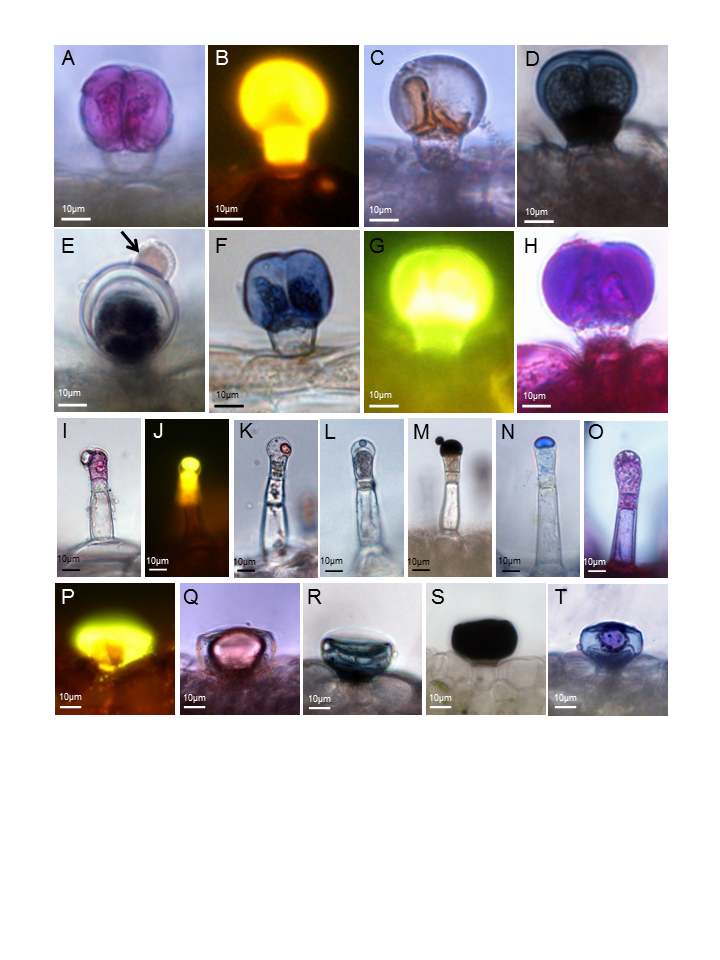

Supplement: Figure S4 — Bright field and fluorescence micrographs of three glandular trichome types showing histochemical characterization of secretory products. (A–H) Histochemistry of the short-stalked capitate glandular trichomes: (A) Ruthenium Red test showing the apical cells stained red; (B) gold-yellow secondary fluorescence observed with Neutral Red under UV light; (C) positive staining reaction with Sudan III in the apical cells and weak reaction in the stalk cell; (D) the apical cells stained blue with Sudan Black B and the stalk cell stained black; (E) OsO4 test showing the apical cells and the droplet (arrow) stained black; (F) NADI staining for terpenes is positive in the apical cells; (G) the apical cells react positively with Naturstoffreagent A; (H) PAS test for polysaccharides in apical cells. (I–O) Histochemistry of the long-stalked capitate glandular trichomes: (I) ruthenium Red test showing the apical cells stained red; (J) yellow staining of secretion in sub-cuticular space with Neutral red; (K) secretory material stained with Sudan III; (L) positive staining reaction with Sudan Black B; (M) black staining of secretion with OsO4, secretory process is visible; (N) secretory material reacts positively for terpenes with NADI; (O) mature trichome reacts positively in PAS test for polysaccharides in the head cell and stalk cell. (P–T) Histochemistry of the peltate glandular trichomes: (P) gold-yellow secondary fluorescence observed with Neutral Red under UV light; (Q) secretory material in the sub-cuticular space positive stained with Sudan III; (R) staining for total lipids with Sudan Black B; (S) positive staining reaction with OsO4 in the head cells and weak reaction in the narrow stalk cell; (T) NADI staining for terpenes is positive in the sub-cuticular space. (TIF) [file pone.0077862.s004.tif]
